# Synergy of Charcoal–Cellulose Fibers for Enhanced COD and TSS Removal From Oily Industrial Wastewater: Experimental Assessment and Statistical Modeling

**DOI:** 10.1002/open.70278

**Published:** 2026-10-07

**Authors:** Taha A. Labani, Rajeev Kumar, Fahed A. Aloufi, M. A. Barakat

**Affiliations:** ^1^ Department of Environment Faculty of Environmental Science King Abdulaziz University Jeddah Saudi Arabia; ^2^ Central Metallurgical R & D Institute Cairo Egypt

**Keywords:** animal charcoal, COD removal, oily wastewater, statistical analysis, tissue paper

## Abstract

This study evaluates a low‐cost fiber medium composed of animal charcoal and tissue paper (AC–TP) as a bench‐scale polishing approach for treating oily industrial wastewater from Makkah Power Plant. Thirty‐six samples were collected over 6 months from three operational areas: the fuel unloading area (FUA), the fuel tank area (FTA), and the turbine unit area (TUA). The samples were analyzed for chemical oxygen demand (COD), total suspended solids (TSS), total dissolved solids (TDS), turbidity, and oil and grease. COD reduction was the primary indicator, while AC–TP‐corrected filter‐retained solids mass and filtration time were supporting indicators. Dose screening on FUA samples with AC:TP ratio of 1.0:0.5 was identified as the preliminary best‐performing dosage based on COD reduction, with corrected filter‐retained solids behavior and available filtration‐time data used as supporting indicators. Applying this dosage to FUA, FTA, and TUA wastewater yielded COD removals of about 75%–82%, 79%, and 79.5%, respectively. Retained solids mass increased by 20%–55%, and filtration time decreased by up to ~60%. Statistical analyses using analysis of variance (ANOVA), paired *t‐*tests, and COD–TSS/COD–filtration‐time regressions confirmed the significance of these improvements. Scanning electron microscope (SEM) and X‐ray diffraction (XRD) observations supported a tentative interpretation of coupled carbon‐assisted organic removal and fiber‐assisted physical retention.

## Graphical abstract

A schematic flow diagram of fuel handling operations and drainage points at Makkah Power Plant. Filtration/Sorption technique for treating oily wastewater from Makkah Power Plant using animal charcoal–tissue paper (AC–TP) composite powdered AC–TP.

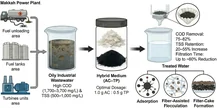

## 1 Introduction

Production, demand, and consumption of electricity are increasing worldwide, and power station networks have expanded rapidly throughout urban areas and industrial centers, including oil‐dependent nations in the GCC region. Oil‐based power plants generate and discharge large volumes of oily wastewater during refueling operations, equipment cleaning, heat management, and safety emergency system testing [1, 2]. The greasy, oily runoff frequently occurs during fuel transfers between tanks during engine maintenance, turbine washing, and fire suppression unit operations at fossil fuel‐burning power plants [3]. The fluid contains three different phases of oil, water, and sludge, which creates problems during separation because it maintains small oil droplets that stay suspended in water for extended periods in aquatic environments [4, 5]. The problem becomes more severe due to two factors: the high volume of waste and the presence of substances such as oil compounds, metal fragments, cleaning products, tiny particles, and chemical additives used for maintenance [6, 7].

The treatment of oily wastewater using gravity separation, dissolved air flotation, coagulation–flocculation, and membrane filtration becomes less effective when dealing with stable emulsions and fine particles. The methods used for free oil and solids management are effective, but they require ongoing chemical supplies, power consumption, and scheduled maintenance, which increase operational expenses [8]. Recent studies have therefore emphasized process intensification, including micro‐/nanobubble‐assisted flotation and optimized coagulant–flocculant systems to improve the capture of fine emulsified oil. Despite membrane filtration achieving significant separation, oil‐induced fouling remains a major obstacle, resulting in increased energy consumption and cleaning frequency; hence, the investigation of next‐generation superhydrophilic/underwater superoleophobic and self‐cleaning membrane surfaces is growing to reduce fouling and maintain fluidity. In tandem with a wider trend toward hybrid and material‐based approaches for the separation of complex oily wastewater, emerging sorbent‐based materials (e.g., cellulose‐derived aerogels) have been proposed for combined oil–water separation and contaminant removal [9, 10, 11, 12]. Researchers are focusing on sustainable methods and processes/processes like adsorbent‐rich or combined filter materials that perform both debris trapping and contaminant absorption in a single process [13]. The powdered animal charcoal (AC) is a common choice because it provides a large surface area and a porous structure, enabling it to absorb multiple organic compounds, including residual hydrocarbons resulting from power plant emissions [14]. The material functions as a standalone solution but fails to prevent pore blockage effectively and to remove small particles from industrial wastewater streams. The use of natural fibrous cellulose can be an effective flocculant and adsorbent material that helps with clumping and structural bonding via charge neutralization and adsorption. Cellulose‐derived materials have been extensively studied in water treatment and oil–water separation, particularly when processed into fibers, films, or modified aerogels. However, untreated cellulose fibers, such as the tissue paper (TP) used in this study, are generally hydrophilic due to their hydroxyl‐rich structure [15]. A combination of cellulosic fibers with AC enables dual contamination removal, as the fibers use chemical absorption to capture dissolved substances and physical trapping to capture solid particles, leading to better oily wastewater purification [16, 17]. This study analyzes an economical filtration/sorption technique using animal charcoal–tissue paper (AC–TP) composite powdered AC–TP for treating oily wastewater from Makkah Power Plant using a condensed AC‍–‍TP composite. This study specifically assesses chemical oxygen demand (COD) reduction, AC–TP‐corrected filter‐retained solids mass, and filtration‐time behavior for oily wastewater from the fuel unloading area (FUA), fuel tank area (FTA), and turbine unit area (TUA). It also analyzes how the AC:TP mass ratio affects bench‐scale treatment performance and discusses structural and morphological changes of the AC–TP medium pre‐ and postfiltration using X‐ray diffraction (XRD) and scanning electron microscope (SEM) to support a cautious interpretation of surface deposition, particulate accumulation, and filter‐cake formation.

## 2 Materials and Methods

### 2.1 Study Site and Sampling Strategy

The study was conducted at the Makkah Power Plant, which uses diesel fuel to generate electricity. Industrial wastewater samples were collected from the FUA, FTA, and TUA. A schematic flow diagram of fuel handling operations and drainage points at Makkah Power Plant is shown in Figure 1. A total of 36 samples were collected over 6 months from June to November 2024. For each zone, two samples per month were collected, totaling 6 per month and 12 per zone across the study period. Sample codes and corresponding collection dates are summarized in Table S1.

**FIGURE 1:**
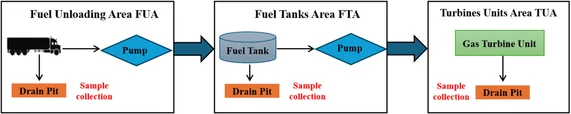
A schematic flow diagram of fuel handling operations and drainage points at Makkah Power Plant.

### 2.2 Chemicals and Materials

The main sorbent was commercial powdered AC (Hopkin & Williams, laboratory reagent grade Cat# 298200), which was employed as the primary sorbent. AC is typically a bone‐char‐type carbon material derived from the carbonization process of animal bones [17]. It usually comprises a fraction rich in carbon and in calcium‐phosphate mineral phases, including components similar to hydroxyapatite. The material was used as is, without washing, sieving, chemical activation, or additional heat treatment. According to the available material specifications, the particle size of the AC powder ranged from approximately 45 to 150 µm, roughly corresponding to the 100–325 mesh based on standard sieve aperture conversions. Brunauer–Emmett–Teller (BET) surface area, pore size distribution, ash content, and hydroxyapatite content were not independently measured in this study; therefore, these parameters are identified as limitations and targets for future characterization.

Waste TP was collected locally and kept under UV light (112 W lamps) for 2 h for disinfection. Thereafter, TP was soaked and crushed in deionized (DI) water using a blender and washed several times with hot and cold DI water. Crushed TP was dried at 105 °C for 24 h and stored for further use.

### 2.3 Instrumentation

Standard analytical procedures were followed according to APHA Standard Methods and USEPA‐approved methods where applicable [18, 19]. pH measurement and conductivity/total dissolved solids (TDS) were measured using a multiparameter electrochemistry meter (Hach HQ4200 with IntelliCAL probes) with pH as a control [20]. The determination of turbidity using a laboratory turbidimeter (Hach 2100N, EPA‐compliant) was based on studies of contaminated water [21, 22] To test COD and nutrient species (ammonia, nitrite, nitrate, and phosphate), precalibrated vial tests (TNTplus) were used (Hach DR6000/DR3900) using UV–vis spectrophotometry, and COD was digested in a digital reactor block (Hach DRB200) [23, 24, 25, 26, 27, 28]. Total suspended solids (TSS) were determined by weight according to standard method 2540 D, which involves filtration, drying at 103–105 °C, and reweighing, using vacuum filtration, a drying oven (e.g., Memmert UN110), and an analytical balance with a reading accuracy of 0.1 mg [18]. Oil and grease were measured as *n*‐hexane extractable material following EPA Method 1664 (gravimetric) [29]. In the treatment and filtration experiments, vacuum filtration was performed using a Rocker 400 oil‐free vacuum pump, equipped with a vacuum gauge/regulator and overflow protection [30]. The manufacturer reported a maximum vacuum value of approximately 99 mbar absolute, equivalent to approximately −680 mmHg, and a flow rate of approximately 34 L per minute at 220 V/50 Hz. For material characterization, XRD was performed using a Panalytical X’Pert PRO 3040/60 (The Netherlands), equipped with Ni‐filtered Cu Kα radiation (*λ* = 0.15406 nm), with an acceleration voltage and current density of 40 kV and 30 mA, respectively. The morphological structures were determined using an SEM instrument obtained from JEOL (Tokyo, Japan).

### 2.4 Removal of the Pollutants From the Oily Wastewater

The removal of contaminants from oily wastewater was tested with AC, TP, and mixtures of AC–TP. For each test, 100 mL of oily wastewater was placed in a lab beaker and mixed with specific amounts of AC and/or TP according to the tested ratio. Then the mixture was stirred for 20 min at 25 °C using a magnetic stirrer at about 300–500 rpm to keep the AC–TP particles dispersed. The pH was checked before treatment but not chemically adjusted; therefore, treatment was performed at the wastewater samples’ natural pH. The mixture was allowed to settle undisturbed for 24 h at laboratory temperature after stirring, to allow aggregation, floc/cake formation, and sedimentation to occur before filtration. A conservative bench‐scale screening condition selected a settling period of 24 h to allow adequate contact, aggregation, and phase clarification prior to vacuum filtration. This duration was not meant to represent optimized industrial hydraulic retention time; shorter settling times should be assessed in future scale‐up studies.

Filtration by vacuum followed, using Whatman Grade 42 quantitative ashless circular filter paper with a nominal diameter of 47 mm and a nominal particle‐retention rating of 2.5 µm. The nominal filtration area is roughly 17.35 cm^2^. Filtration took place via the Rocker 400 oil‐free vacuum pump previously described in Section 2.3. During experiments, the built‐in regulator controlled the vacuum to maintain stable laboratory suction. The filtrate was collected for COD analysis.

The used filter paper was dried at 103–105 °C and weighed again to determine how much solid it had retained during filtering. Since some of the added AC–TP medium might remain on the filter after filtering, an AC–TP material blank was prepared for each dosage using DI water instead of wastewater. Each blank received treatment under identical AC–TP mass, stirring conditions, settling time, vacuum‐filtration conditions, drying temperature, and weighing method as its corresponding wastewater‐treated sample, thus enabling accurate comparison between blanks and samples. The increase in mass recorded in the AC–TP material blank corresponded to the retained mass of the treatment medium itself, and this value was deducted from the measured increase in mass for the sample treated with wastewater. So, what is reported as retained solids value is actually net filter‐retained solids mass related to wastewater‐derived solids corrected by AC–TP correction factor.

The corrected retained mass was calculated as follows:

$$M_{\mathrm{net}}=\Delta M_{\mathrm{sample}}-\Delta M_{\mathrm{blank}}$$

where *M*_net_ is the net solids mass retained by the filter corrected for AC–TP, Δ*M*_sample_ is the increment in filter mass after it has been used to filter and dry a sample of wastewater treated with AC–TP, and Δ*M*_blank_ is the increment in filter mass obtained from the corresponding material blank for AC–TP prepared with DI water under identical experimental conditions. Each Δ*M* value was calculated as dried filter mass after filtration minus initial clean filter mass before filtration.

Reagent blanks and instrument calibration necessary for chemical analyses were done according to the requirements outlined in the analytical method. Filtration without AC–TP served as the operational control for comparing corrected retained solids mass and filtration‐time behavior.

To identify a preliminary best‐performing treatment, the most effective treatment dosage was determined by testing a series of AC and TP combinations on wastewater samples. The initial experiments were conducted on samples collected from the FUA, where different AC:TP ratios were evaluated to assess their effect on COD reduction performance (Table S2).

After filtration and drying, the spent AC–TP medium on the filter paper was considered potentially contaminated solid waste due to possible inclusion of suspended solids derived from wastewater and organic residues associated with oil. The used material was collected in specifically marked contaminated‐waste containers and disposed of in accordance with the laboratory's approved waste management procedure.

### 2.5 Statistical Analysis

Analyses were conducted in IBM SPSS Statistics (v26; IBM Corp., Armonk, NY, USA). Data are reported as mean ± SD, and Pearson correlations were used to assess associations among COD, TSS, turbidity, and filtration time. The effect of AC:TP dosage on COD removal in the FUA was tested using one‐way analysis of variance (ANOVA) with Tukey's honestly significant difference (HSD), and COD removal at the preliminary best‐performing dosage was compared across the three wastewater sources using one‐way ANOVA. Since the dosage‐screening dataset was limited, the dosage analysis was interpreted as exploratory support rather than as fully confirmatory optimization. Pre‐ versus post‐treatment differences in COD, AC–TP‐corrected filter‐retained solids mass, and filtration time were evaluated with paired‐samples *t*‐tests; effect sizes were reported as Cohen's d (*t*‐tests) and eta‐squared (*η* ^2^) (ANOVA). Simple linear regression was applied to model COD–TSS (baseline pretreatment dataset) and COD–filtration time (preliminary best‐performing dosage subset). Assumptions were checked using Shapiro–Wilk (normality) and Levene (homogeneity), with *p *< 0.05 as the significance threshold. The statistical equations used are summarized in Table 1.

**TABLE 1:** Statistical equations used in the study.

Statistics/test	Equation (text form)	Where/notes
Mean	$\bar{x}$ = (Σ *x*_*i*)/*n*	*x*_*i*: observation *i*; *n*: sample size.
Standard deviation (SD)	SD = sqrt( Σ (*x*_*i* − $\bar{x}$)^2/ (*n*−1))	Sample SD (denominator *n*−1).
Percent removal	%*R *= ((*C*0−*C*t)/*C*0) × 100	*C* 0: pretreatment concentration; *C*t: post‐treatment concentration.
Pearson correlation	*r *= Σ[(*x*_*i* − $\bar{x}$)(*y*_*i* − $\bar{y}$)]/sqrt(Σ(*x*_*i* − $\bar{x}$)^2×Σ(*y*_*i*−$\bar{y}$)^2)	Two‐tailed *p*‐value computed in SPSS.
Paired‐samples *t*‐test	*t* = $\bar{d}$/(SD_*d*/sqrt(*n*))	*d*_*i *= (pre_*i*−post_*i*); $\bar{d}$: mean of paired differences; SD_*d*: SD of differences.
Cohen's *d* (paired)	*d*_*z *= $\bar{d}$/SD_*d* (equivalently: *d*_*z *= *t*/sqrt(*n*))	Reported as Cohen's *d* for paired comparisons.
One‐way ANOVA	*F* = MS_between/MS_within	MS_between = SS_between/(*k*−1); MS_within = SS_within/(*N*−*k*).
Eta‐squared (ANOVA effect size)	*η* ^2^ = SS_between/SS_total	Proportion of total variance explained by the factor.
Tukey's HSD (post hoc)	HSD = *q*_(*α*,*k*,df) × sqrt(MS_within/*n*_*g*)	*q*: studentized range critical value; *n*_*g*: per‐group *n* (balanced case). Computed in SPSS.
Simple linear regression (model)	*y *= *β*0 + *β*1 *x *+ *ε*	*x*: predictor; *y*: response; *ε*: error term.
Shapiro–Wilk (normality)	*W *= ( (Σ *a*_*i x*_(*i*)) ^2)/Σ(*x*_*i*−$\bar{x}$) ^2	*x*_(*i*): ordered sample values; *a*_*i*: constants; computed in SPSS.
Levene's test (homogeneity)	*F *= [(*N*−*k*)/(*k*−1)] × [ Σ *n*_*j* ($\bar{Z}$_*j* −$\bar{Z}$)^2]/[ Σ Σ (*Z*_ij − $\bar{Z}$_*j*)^2]	*Z*_ij = |*Y*_ij − $\tilde{Y}$_j|; computed in SPSS.

## 3 Results and Discussion

### 3.1 Physical and Chemical Analysis of Oily Wastewater

The physicochemical characteristics of the collected oily wastewater samples from different operational zones in the power plant are tabulated in Tables 2, 3, 4. Summary descriptive statistics (mean ± SD; *n* = 12 per area) are provided in Table S3. The measured parameters included pH, TDS, tTSS, turbidity, COD, oil and grease, ammonia (NH_3_–N), nitrite (NO_2_^−^), nitrate (NO_3_^−^), and phosphate (PO_4_^3−^). Standard procedures were followed in accordance with the American Public Health Association (APHA, 2023) [18] and United States Environmental Protection Agency (USEPA) [19] methods. These measurements were used to establish the baseline wastewater profile for the three operational areas. For the treatment phase, COD reduction was the primary performance indicator, while AC–TP‐corrected filter‐retained solids mass and filtration‐time behavior served as supporting operational indicators.

**TABLE 2:** Characteristics of wastewater samples collected from the fuel unloading area (FUA).

Code	pH	TDS, mg/L	TSS, mg/L	Turbidity, NTU	COD, mg/L	Ammonia, mg/L	Nitrite, mg/L	Nitrate, mg/L	Phosphate, mg/L	Oil/grease, mg/L
FUA1	7.9	206	718	660	2591	0.56	1.33	13.9	0.67	388.65
FUA2	6.8	249.4	742	680	2671	1.0	0.55	10.22	1.78	400.65
FUA3	6.25	263	769	720	2723	0.41	2.75	12.7	2.3	408.45
FUA4	7.5	207.9	715	660	2572	1.21	0.93	13	1.2	385.8
FUA5	7.7	190.4	669	650	2268	0.86	1.17	6.1	2.91	340.2
FUA6	6.8	192.1	619	620	2055	0.06	2.33	7.8	1.36	308.25
FUA7	6.3	166	567	560	1919	1.1	1.6	8.63	2.42	287.85
FUA8	7.1	160.4	641	640	2142	0.9	1.33	11	0.9	321.3
FUA9	6.0	183	630	600	2192	0.8	1.74	13.6	1.21	328.8
FUA10	7.4	194	690	670	2371	0.81	1.3	10.1	2.21	355.65
FUA11	7.6	181	672	650	2288	0.3	1.1	10.8	1.69	343.2
FUA12	6.7	159	622	590	2156	0.1	2.3	14.6	1.4	323.4

**TABLE 3:** Characteristics of wastewater samples collected from the fuel tank area (FTA).

Code	pH	TDS, mg/L	TSS, mg/L	Turbidity, NTU	COD, mg/L	Ammonia, mg/L	Nitrite, mg/L	Nitrate, mg/L	Phosphate, mg/L	Oil/grease, mg/L
FTA1	6.9	316	1013	910	3721	1.58	1.56	15	0.15	558.15
FTA2	7.8	296	985	860	3681	0.92	1.12	8.87	2.24	552.12
FTA3	7.25	277	948	810	3598	1.3	3.8	14.0	0.79	539.7
FTA4	6.6	289	967	830	3613	1.67	2.2	11.36	1.44	541.95
FTA5	7.0	227	904	790	3385	0.56	1.61	3.5	3.54	507.75
FTA6	7.6	190.6	851	770	3087	0.42	2.0	4.89	1.96	463.05
FTA7	7.2	207	887	800	3241	0.87	2.6	6.3	3.02	486.15
FTA8	6.1	214	908	810	3341	1.1	1.9	16	0.44	501.15
FTA9	7.8	182	849	750	3153	1.56	1.4	15.3	1.76	472.95
FTA10	6.5	203	948	820	3593	1.89	2.3	9.63	2.69	538.89
FTA11	6.8	299	974	860	3621	1.46	1.8	12.6	2.81	543.15
FTA12	6.3	264	927	810	3473	1.29	2.7	17	0.6	520.67

**TABLE 4:** Characteristics of wastewater samples collected from turbine unit area (TUA).

Code	pH	TDS, mg/L	TSS, mg/L	Turbidity, NTU	COD, mg/L	Ammonia, mg/L	Nitrite, mg/L	Nitrate, mg/L	Phosphate, mg/L	Oil/grease, mg/L
TUA1	6.88	139	553	540	1868	0.32	0.24	6.94	0.15	280.2
TUA2	7.95	145	573	570	1907	0.97	0.36	5.38	2.24	286
TUA3	6.63	121	562	550	1892	1.1	0.46	3.34	0.79	283.8
TUA4	7.4	173	601	600	1986	0.18	0.12	2.05	1.44	298
TUA5	7.6	187	634	640	2089	0.25	0.27	5.09	3.54	313.35
TUA6	7.3	198	662	680	2151	0.22	0.71	1.4	1.96	322.65
TUA7	6.8	155	589	590	1961	0.4	0.48	7.51	3.02	294.15
TUA8	6.9	131	518	500	1782	0.21	0.49	8.32	0.44	267.3
TUA9	7.5	149	540	530	1813	0.32	0.01	6.65	1.76	271.9
TUA10	7.1	152	589	600	1923	0.19	0.21	4.98	2.69	280.2
TUA11	7.8	138	571	570	1877	0.3	0.32	8.9	2.81	286.23
TUA12	7.17	123	505	480	1744	0.23	0.05	10	0.6	283.8

Descriptive statistics (mean ± standard deviation) calculated for each operational area further emphasized these contrasts. The FTA exhibited the highest average pollution levels, with mean COD, TSS, and oil and grease concentrations of 3458.92 ± 214.12, 930.08 ± 51.95, and 518.81 ± 32.11 mg/L, respectively. In comparison, the TUA showed the lowest baseline contamination, with mean COD, TSS, and oil and grease values of 1916.08 ± 118.61, 574.75 ± 44.89, and 288.96 ± 16.02 mg/L, respectively. The FUA displayed intermediate conditions, with mean COD, TSS, and oil and grease concentrations of 2329.00 ± 258.30, 671.17 ± 58.51, and 349.35 ± 38.74 mg/L, respectively. Based on the results in Tables 2–4, samples collected from the FTA demonstrated the highest level of contamination followed by FUA and TUA. This could be due to chronic contamination sources in the FTA, including the accumulation and periodic removal of tank‐bottom sludges (hydrocarbons–water–sediment mixtures) which can increase organic load (COD) and carry suspended solids and oil residues compared with the unloading and turbine areas.

### 3.2 Treatment of Oily Wastewater

The effectiveness of the AC, TP, and mixed dose of AC–TP on the COD reduction, AC–TP‐corrected filter‐retained solids mass, and filtration time from the FUA oily wastewater samples was investigated and the results are summarized in Table 5. The results in Table 5 demonstrated that a mixture of 1.0 g AC and 0.5 g TP provided the highest COD removal, with efficiencies of 82%, 75.2%, and 78.9% for FUA2, FUA6, and FUA12, respectively. The dose–response trend is shown in Figure S2, and the overall dosage effect was statistically significant in the exploratory one‐way ANOVA (*p *= 0.011; Table S5), with key pairwise differences supported by Tukey's post hoc comparisons (Text S1). However, since the dosage‐screening dataset was limited, these statistical results are interpreted as supportive evidence for dose selection rather than as a fully confirmatory optimization.

**TABLE 5:** Performance indicators at different AC:TP dosage ratios for FUA wastewater samples.

Sample source	Dosage as AC:TP, g	% COD reduction	Retained solids mass change (with vs. without AC–TP), %	Filtration‐time reduction (with vs. without AC–TP), %
FUA2	1:0	65.03	19	NA
	0.5:0	48.67	39.29	NA
	0.25:0.25	52.08	19.35	NA
	1:0.25	71.92	15.22	NA
	1:0.5	82	28	60.62
FUA6	0:1	15.04	12	NA
	0.5:0.5	52.51	17.07	NA
	1:0.5	75.2	31	63.07
	1:0.25	62.04	12.77	NA
FUA12	1:1	75	30.77	NA
	0.25:0	34.65	26	NA
	1:0.5	78.9	25	62.46
	1:0.25	56	19	NA

The superior performance at higher AC contents can be attributed to the increased adsorptive surface area and binding sites of the carbon, which enhance the removal of dissolved organic compounds. At the same time, the cellulosic tissue fibers promote flocculation and physical entrapment of suspended solids. The weaker performance at low dosages (e.g., 0.25:0.25 or 0.5:0.5) may reflect the insufficient availability of active adsorption sites. Based on the results outlined in Table 5, the 1.0 g AC:0.5 g TP ratio was chosen as the preliminary best‐performing dosage under the conditions of this bench‐scale screening study. This choice was made primarily based on COD reduction and supported by the behavior of AC–TP‐corrected filter‐retained solids and available filtration‐time data. It should be noted that filtration‐time reduction was not available for all dosage ratios in the screening dataset; thus, filtration‐time behavior could only be used as a supporting operational indicator where measurements were available. This preliminary selected dosage was then tested on three representative samples from both the FTA and TUA, yielding average COD removal efficiencies of 78.6% and 79.4%, respectively, as summarized in Table 6.

**TABLE 6:** Performance indicators at different AC:TP ratios for FTA and TUA wastewater samples.

Sample source	Dosage as AC:TP, g	% COD reduction	Retained solids mass change (with vs. without AC–TP), %	Filtration‐time reduction (with vs. without AC–TP), %
FTA2	1:0.5	78.57	21.05	58.65
FTA6	1:0.5	78.81	31.67	55.41
FTA12	1:0.5	79	22.22	54
TUA2	1:0.5	79	36.36	22.06
TUA6	1:0.5	77.73	26.83	26.09
TUA12	1:0.5	78.15	54.55	59.66

With the same preliminary selected dosage, AC–TP‐corrected filter‐retained solids mass also increased for the FUA samples listed in Table 5. The changes in retained solids mass relative to filtration without treatment material were 28% for FUA2, 31% for FUA6, and 25% for FUA12, indicating enhanced retention of wastewater‐derived particulates and associated residues by the AC–TP layer. The available data on filtration time in Table 5 showed improved filtration performance, with reductions of 60.62% for FUA2, 63.07% for FUA6, and 62.46% for FUA12. A summary comparison of pre‐ and post‐treatment performance at the preliminary selected dosage across operational areas is provided in Figure S3 of the Supporting Information. The observed trends are consistent with previous studies on oily and hydrocarbon‐contaminated wastewaters, where AC‐based media achieved substantial COD reductions and improved treatment robustness through coupled adsorption‐flocculation mechanisms [31, 32, 33, 34]. Likewise, the enhanced capture of suspended matter and improved filtrate clarity align with reports showing that carbon‐based and fibrous/cellulose‐supported composite materials can strengthen solid–liquid separation by promoting aggregation and physical entrapment of particulates [35, 36, 37, 38, 39, 40, 41, 42]. In addition, the reductions in filtration time are in agreement with studies demonstrating improved filter‐cake structure, permeability, and flow rate when precoat layers or composite adsorbent media are applied in oily and industrial wastewater filtration systems [43, 44, 45, 46, 47, 48]. The results support that 1:0.5 AC:TP dosage is an effective compromise between pollutant removal efficiency and material utilization. These results indicate that the selected AC–TP dosage consistently achieves high COD reductions across all three wastewater sources at the Makkah Power Plant.

The influence of AC–TP dosage on COD removal in the FUA was evaluated by one‐way ANOVA. After verifying that the assumptions of normality (Shapiro–Wilk, *p *= 0.215) and homogeneity of variances (Levene, *p *= 0.794) were satisfied, the ANOVA indicated a significant effect of dosage on COD removal efficiency, with *F*(8, 4) = 14.14 and *p *= 0.011. The corresponding eta‐squared (*η* ^2^) value of 0.966 indicated a large apparent dosage effect within the screening dataset (Table S5). Tukey's HSD test showed that the mixed dosage (1.0 g AC:0.5 g TP) achieved COD removals that were 63.4% higher than TP alone (0 g AC:1.0 g TP; *p *= 0.008) and 43.8% higher than a low‐carbon dosage (0.25 g AC; *p *= 0.030), whereas no significant difference was observed compared with 1.0 g AC:1.0 g TP (difference 3.4%; *p *= 0.999), indicating that additional tissue beyond 0.5 g does not provide further COD removal benefits (Text S1). Effect sizes, including *η* ^2^ for ANOVA and Cohen's *d* for paired‐samples *t*‐tests, were reported to provide additional context for the magnitude of the observed treatment effects. At the preliminary selected dosage (1.0 g AC:0.5 g TP), COD removal remained consistently high across the other wastewater sources. In the FTA, influent COD levels of 3087–3681 mg/L decreased to 654–789 mg/L, corresponding to removal efficiencies of approximately 78%–79%. In the TUA, influent COD levels of 1744–2151 mg/L were reduced to 381–479 mg/L, yielding comparable removal efficiencies of 77%–79%. When all areas were pooled, paired‐samples *t*‍‐‍tests supported significant pre‐ to post‐treatment changes in COD, filter‐retained solids mass, and filtration time, with large effect sizes (Table S6). Analysis of the filter‐retained mass in FUA showed a consistent increase after applying AC–TP; at the preliminary selected ratio (1.0 g AC:0.5 g TP), corrected retained mass increased from 0.050 g (no material) to 0.064 g (with material), corresponding to a pooled increase of 0.012 g (pre–post mean difference = −0.012 g; Table S6). At the preliminary selected dosage, removal efficiency did not differ significantly among operational areas (one‐way ANOVA; *p *= 0.937; Table S7). Table 7 summarizes the pre‐ and post‐treatment means, showing a marked decrease in COD and filtration time, alongside an increase in AC–TP‐corrected filter‐retained solids mass across the three operational areas.

**TABLE 7:** Descriptive statistics (means) for the pre‐ and post‐treatment conditions across the three areas.

Source	Variable	Pretreatment mean	Post‐treatment mean
Fuel unloading area	COD, mg/L	2294.00	489.33
	Retained solids, g	0.046	0.058
	Filtration time, s	32.2	12.2
Fuel tank area	COD, mg/L	3413.67	725.00
	Retained solids, g	0.063	0.079
	Filtration time, s	107.3	46.9
Turbine unit area	COD, mg/L	1934.00	421.00
	Retained solids, g	0.032	0.044
	Filtration time, s	38.9	26.1

#### 3.2.1 Linear Regression Modeling Between COD, TSS, and Filtration Time

Beyond the area‐based comparisons, simple linear regression models were developed to quantify the relationships between COD, TSS, and filtration time, as summarized in Figure 2. Because the correlation between COD and TSS was strong (*r* = 0.995), the correlation between COD and TSS was extremely strong (*r* = 0.995; Table S4 and Figure S1). A regression model was fitted using the full dataset (*n* = 36). As illustrated in Figure 2a, the resulting equation:

**FIGURE 2:**
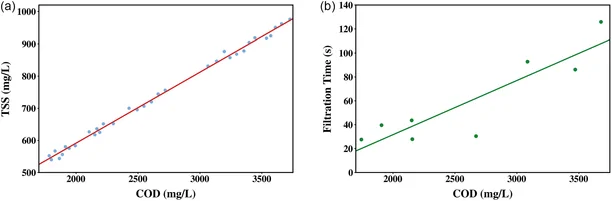
Linear regression relationships between (a) COD and total suspended solids (TSS) and (b) COD and filtration time for the wastewater samples from the three operational areas.

TSS (mg/L)=0.2313×COD (mg/L)+131.35

The relationship between COD and TSS best fits a linear model, with *R* ^2^ = 0.993 and a *p*‐value <0.001. This indicates that COD and TSS are closely related in the wastewater samples collected, thereby reflecting a strong relationship between organic load and suspended solids in all three plant areas from which samples were taken. Therefore, it can be concluded that COD might be an appropriate operational indicator of pollution load for this particular dataset. However, more generally applicable uses of COD as a surrogate for TSS will need further validation with independent sample sets.

A second regression model was used to explore how organic load affects process efficiency by relating COD to filtration time for the subset of experiments conducted at the preliminary best‐performing AC–TP dosage (*n* = 9). As shown in Figure 2b, the fitted equation:

Filtration time (s)=0.048×COD (mg/L)−62.91

The relationship between COD and filtration time was described by an equation with *R* ^2^ = 0.810 (*p *< 0.001). It implies that under the bench‐scale conditions tested, samples with higher COD more often required longer filtration times. On average, a 100 mg/L increase in COD was estimated to increase filtration time by about 4.8 s. This finding supports the interpretation that organic load influences AC–TP treatment filtration behavior; however, further validation across different operating conditions would be needed to fully confirm this effect.

Data points represent individual samples, and solid lines indicate the fitted linear regression models.

#### 3.2.2 Comparative Performance Assessment

The performance of COD removal with the present AC–TP medium is compared with representative low‐cost adsorbents, activated‐carbon systems, cellulose‐based filters, biochar, commercial filtration media, membrane‐based pretreatment systems, and coagulation/electrocoagulation processes reported for oily wastewater treatment in Table 8. Relative significance and novelty of the AC–TP medium are highlighted through Table 8. Direct comparisons should be taken with caution, as wastewater composition, pollutant concentrations, treatment configurations, contact/settling times, and analytical endpoints vary from one study to another.

**TABLE 8:** Summary of quantitative and practical comparisons of AC–TP with some selected methods for treating oily wastewater.

Material/process	COD removal/related endpoint	Cost‐complexity/practical limitation	Position relative to AC–TP	References
AC–TP medium	COD removal: ~75%–82% for FUA and ~79% for FTA/TUA	Low material complexity; commercial animal charcoal + untreated tissue paper. Limitation: 20 min stirring + 24 h settling + vacuum filtration; bench scale only	Simple preliminary polishing medium combining carbon‐assisted COD reduction and fiber‐assisted retention	This study
Activated carbon/low‐cost adsorbents	Oil‐sludge‐derived activated carbon: up to ~94% treatment efficiency; water‐hyacinth activated carbon: 54.65% COD removal from oil/gas wastewater	Medium–high: carbonization, activation, and characterization are usually required	Can achieve higher removal, but the preparation is more complex than using commercial animal charcoal directly	[49, 50]
Cellulose‐based filters/modified cellulose membranes	Modified cellulose‐acetate filters: COD cleanup up to 99%; bacterial cellulose membranes: ~99% oily‐contaminant removal; endpoints vary	Medium–high: membrane fabrication, biological production, nanocellulose processing, or hydrophobic/surface modification often required	Higher performance cellulose systems exist; AC–TP uses untreated hydrophilic tissue mainly as support, not modified hydrophobic cellulose	[51, 52, 53]
Biochar/modified biochar	Magnetic palm‐shell biochar: 87.91% COD, 88.46% TSS, and 99.96% oil/grease removal from greywater; KOH‐biochar showed strong COD adsorption behavior	Medium: feedstocks may be low‐cost, but pyrolysis, activation, magnetic modification, and characterization increase complexity	Useful carbon benchmark; AC–TP is simpler but should not claim superiority to engineered biochars	[54, 55]
Commercial filtration media and membrane‐based pretreatment systems	Diatomite/carbon media: ~98%–99% oil rejection; integrated Fenton–AC–UF reduced effluent COD to <50 mg/L; nanofibrous membranes show high oil removal	Medium–high to high: engineered media/membranes require fabrication, flux control, fouling management, cleaning, and regeneration/backwashing	Higher performance filtration benchmark; AC–TP is lower in complexity but less hydraulically validated	[45, 56, 57, 58]
Coagulation/flocculation and electrocoagulation	Optimized FeCl_3_ coagulation: 80.42% COD reduction; electrocoagulation: >90% COD removal and high oil/grease removal under optimized conditions	Medium–high: chemicals or electricity, pH/process control, sludge management, and electrode management may be required	Often more effective under optimized conditions, but more operationally complex than AC–TP	[59, 60, 61, 62]

In general, AC–TP offers COD removal comparable to that reported for some low‐cost adsorbents and coagulation‐based methods; however, engineered cellulose filters, modified biochars, electrocoagulation, and membrane/filtration systems under optimized conditions can achieve higher removals. These systems generally require greater preparation or operational complexity, including activation, membrane fabrication, electrical input, flux control, fouling management, and regeneration. Therefore, AC–TP should be regarded as a low‐complexity bench‐scale polishing medium rather than an alternative to advanced treatment systems. Further work needs to look at shorter settling times, continuous‐flow operation, post‐treatment oil and grease, final effluent TSS, hydraulic loading, and spent‐media regeneration/disposal.

### 3.3 Oily Wastewater Purification Mechanism

The behavior of removal by the AC–TP medium can be understood from the properties of the oily wastewater and both treatment elements together. At its baseline characterization, the wastewater showed a high organic load, suspended solids, turbidity, and oil/grease. This indicated that dispersed oil‐associated and particulate contaminants were present in the sample [63]. The results of treatment indicated COD reduction and corrected filter‐retained solids performance were achieved with the combined dosage‐screening tests using the AC–TP medium than with each component used alone. This is attributed to complementary roles played by AC and TP fibers rather than to a single removal pathway.

It is anticipated that AC will provide most of the carbonaceous adsorption sites for dissolved and colloidal organic constituents. Organic removal may be due to hydrophobic association, van der Waals interactions, pore filling, and possible π–π interactions with carbonaceous domains containing aromatic constituents [64]. The tissue‐paper fibers used herein are cellulose fibers because they are hydrophilic due to their hydroxyl‐rich structure. Their function should hence mainly be physical and structural: the provision of an available fibrous network capable of supporting particle bridging, aggregation, filter‐cake formation, retention of suspended solids, and AC‐associated aggregates during filtration [65, 66]. Therefore, it is postulated that the medium AC–TP operates through coupled carbon‐assisted organic removal plus fiber‐assisted physical retention.

SEM and XRD analyses were applied to aid in interpreting surface deposition, pore coverage, particulate accumulation, and structural changes on the AC–TP medium before and after contact with wastewater. These techniques offer morphological and crystalline‐phase evidence but do not directly confirm specific molecular‐level interactions. In this case, possible π–π interactions and hydrophobic association are mostly related to the carbonaceous fraction of AC; however, hydrogen bonding may take place between oxygen‐containing groups on AC and hydroxyl groups on cellulose fibers. Hence, SEM plus XRD results will be interpreted here more as supportive evidence consistent with pollutant deposition and filter‐cake formation, rather than as conclusive proof of the entire removal mechanism. The surface morphologies of the AC–TP medium before and after treatment at different magnifications are shown in Figure 3. The fibrous cellulosic structure of TP before treatment is illustrated in Figure 3a. The pristine AC–TP medium is shown in Figure 3b,c, where AC particles are found on and between the tissue‐fiber surfaces. A rough, heterogeneous morphology was observed in the pristine AC–TP blend, with visible interparticle/fiber voids; these might provide pathways for contact between wastewater constituents and the AC–TP medium.

**FIGURE 3:**
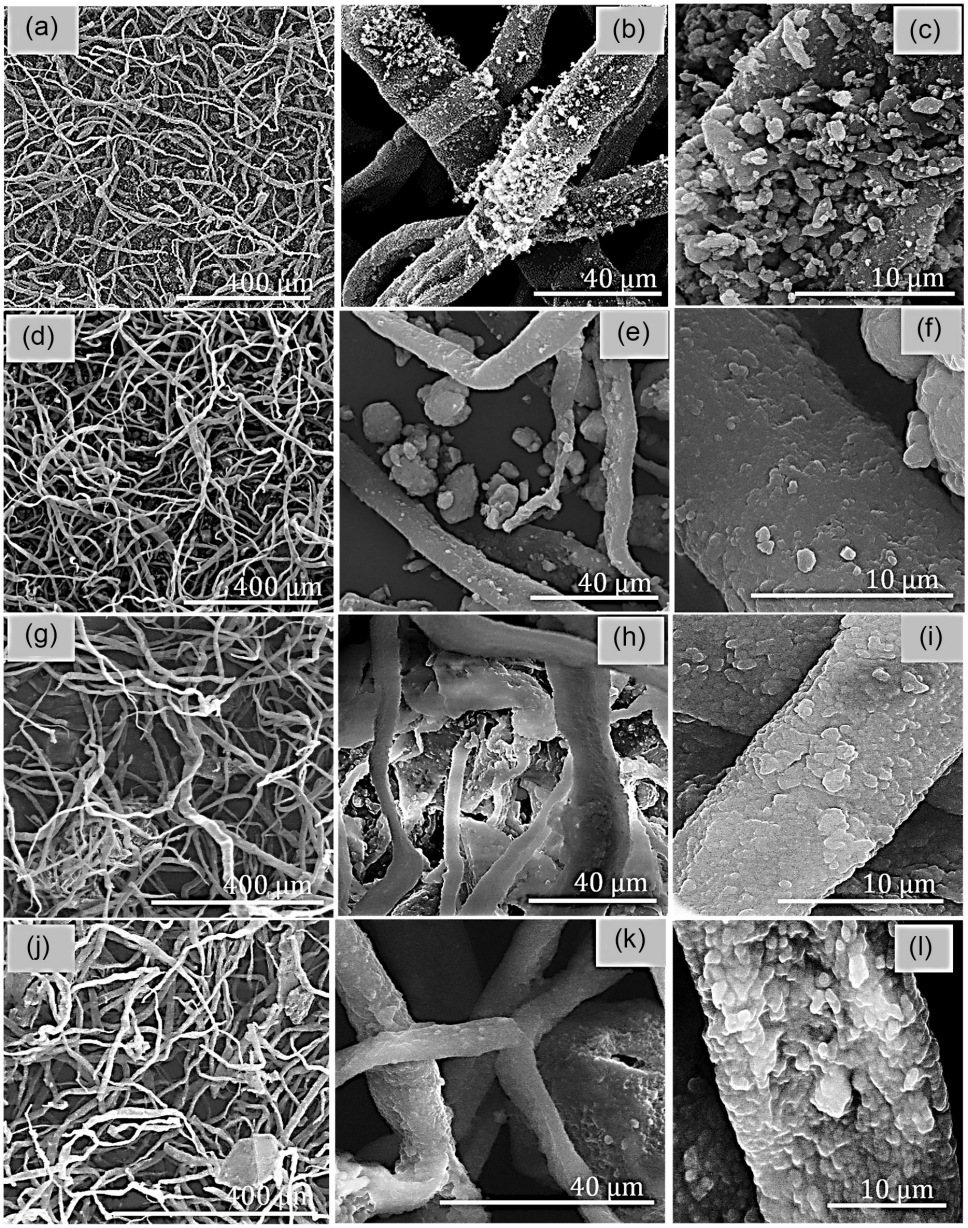
SEM micrographs of the AC–TP before and after oily wastewater treatment: (a–c) pristine AC–TP, (d–f) AC–TP images after treatment of FUA wastewater, (g–i) AC–TP images after treatment of FTA wastewater, and (j–l) AC–TP images after treatment of TUA wastewater.

Figure 3d–f reveals that after treatment with FUA wastewater, the surface of AC–TP became covered with irregularly deposited material and particulate agglomerates, which means that the medium retained particulates derived from wastewater plus organic‐associated residues. In the FTA‐treated medium, Figure 3g–i shows more compact deposited layers with partial coverage of surface voids; this observation agrees with the higher initial COD and suspended solids load in FTA wastewater. The TUA‍‐‍treated AC–TP medium shown in Figure 3j–l has less dense surface coverage, which agrees with a lower baseline organic and particulate load in TUA wastewater. Overall, these observations support the interpretation that the AC–TP medium contributes to physical retention, surface deposition, and filter cake formation during treatment.

XRD analysis was conducted to compare the crystalline and semicrystalline characteristics of TP, pristine AC–TP, and spent AC–TP media after filtration from wastewater. The diffraction patterns were used to assess whether the original cellulose/carbon/mineral background was retained and whether any changes in mineral‐related reflections or peak intensities occurred after treatment. Since XRD does not directly identify adsorbed organic functional groups or confirm molecular adsorption interactions, its results are interpreted as supportive evidence of retained mineral phases and changes consistent with surface/particulate deposition rather than definitive proof of new compound formation. The XRD patterns before and after treatment with oily wastewater are given in Figure 4.

**FIGURE 4:**
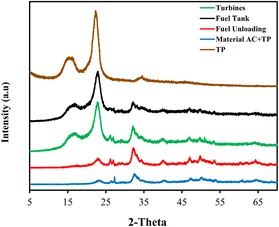
XRD diffraction patterns of pristine tissue paper (TP) and the AC–TP material before treatment and after filtration of wastewater from the fuel unloading, fuel tank, and turbine areas.

The pure AC, TP, and mixed AC–TP dosage‐screening tests show complementary behavior between the carbonaceous and fibrous components. The present study did not quantitatively separate individual contributions of adsorption, sedimentation, filtration, and fiber‐assisted aggregation. Therefore, the proposed mechanism is interpreted as an integrated removal pathway rather than a quantified contribution of each individual process. Future work should include expanded control experiments such as AC alone, TP alone, settling alone, filtration alone, and sequential combined controls to quantify the contribution of each process.

The XRD patterns in Figure 4 compare TP (brown), the pristine filtration medium before wastewater contact, Material AC + TP (blue), and the spent media recovered after filtration of oily wastewater from fuel unloading (red), fuel tank (black), and turbines (green). The TP diffractogram shows the characteristic cellulose‐I reflections, with prominent features in the ~15°–17°, ~22°–23°, and ~34°–35° 2*θ* regions, consistent with the semicrystalline structure of the cellulosic fibers [67]. The pristine AC–TP pattern shows a broad, diffuse contribution in the approximately 20°–30° 2*θ* region, commonly associated with disordered carbonaceous materials, together with weaker mineral‐related reflections consistent with the calcium‐phosphate/apatite‐like mineral fraction expected in animal/bone‐derived charcoal.

After filtration, the spent AC–TP media retained the overall cellulose/carbon/mineral background, indicating that no major transformation of the underlying AC–TP structure occurred during treatment. However, changes in relative peak intensity and the appearance or enhancement of apatite‐consistent reflections, particularly in the approximately 31°–33° and 46°–50° 2*θ* regions, were observed in the spent media [68, 69]. These changes are consistent with the retention of fine inorganic particles and associated mineral deposits on or within the filtration medium during wastewater contact, rather than definitive evidence of new compound formation or direct molecular adsorption. Differences in peak sharpness and relative intensity among the FUA‐, FTA‐, and TUA‐treated media likely reflect variations in particulate/mineral loading and deposition behavior across the different wastewater sources [70]. Therefore, the XRD results provide complementary evidence supporting the SEM‐based interpretation of particulate deposition and filter‐cake formation, but they should not be interpreted as conclusive proof of molecular‐level adsorption mechanisms.

## 4 Conclusions

Herein, bench‐scale polishing tests were conducted with an AC–TP medium on oily industrial wastewater from various operational zones of a power plant. Physicochemical characterization of baseline samples revealed that COD, suspended solids, turbidity, and oil/grease are major pollution indicators, with differences among the three wastewater sources. The preliminary best‐performing AC–TP dosage during dosage screening was chosen at a ratio of 1.0 g AC:0.5 g TP, as it yielded the maximum COD reduction and was confirmed by corrected filter‐retained solids and filtration time behavior, where applicable. At this dosage, COD removal ranged from about 75% to 82% for samples from FUA and was around 79% for both FTA and TUA sample readings. The combined results suggest that the AC–TP medium functions through complementary contributions from both components. The fraction of AC likely provides carbonaceous sites for the removal of dissolved and colloidal organic constituents, while the untreated hydrophilic tissue‐paper fibers mainly act as a fibrous support, promoting particle bridging, surface deposition, filter‐cake formation, and physical retention during filtration. The AC–TP medium demonstrated encouraging bench‐scale performance as a low‐complexity polishing agent for COD reduction and better filter‐retained solids behavior in oily industrial wastewater.

## Funding

This work was supported by KAU Endowment (WAQF) at King Abdulaziz University.

## Conflicts of Interest

The authors declare no conflicts of interest.

## Supporting information

Supplementary Material

## Data Availability

The data that support the findings of this study are available in the supporting information of this article.
