# Supplementary material for: Synergy of Charcoal–Cellulose Fibers for Enhanced COD and TSS Removal From Oily Industrial Wastewater: Experimental Assessment and Statistical Modeling
Source: ChemistryOpen. 2026 Oct 7;15(10):e70278. doi: 10.1002/open.70278 (PMC13645059; doi:10.1002/open.70278)
Supplement: Supplementary file 1 [file OPEN-15-e70278-s001.pdf]

# Supporting Information

## Synergy of charcoal– cellulose fibers for enhanced COD and TSS removal from oily industrial wastewater: Experimental assessment and statistical modelling

**Taha A. Labani[a], Rajeev Kumar[a], Fahed A. Aloufi[a], M.A. Barakat[a,b]***

[a] Department of Environment, Faculty of Environmental Science, King Abdulaziz University, Jeddah-21589, Saudi Arabia
[b] Central Metallurgical R & D Institute, Helwan 11421, Cairo, Egypt

*Corresponding Author; **M.A. Barakat**

Email: mababdullah1@kau.edu, mabarakat@gmail.com

ORCID: 0000-0001-6244-8394

T: +9660126400000

This Supporting Information provides additional statistical outputs and visualizations that complement the main manuscript.

Abbreviations: FUA = Fuel Unloading Area; FTA = Fuel Tanks Area; TUA = Turbine Units Area.

## S1. Sampling design and experimental dosages

**Table S1. Sample identification and collection dates**

| Sampling date | Fuel Unloading Area | Fuel Tanks Area | Turbine Unit Area |
|---|---|---|---|
| 02/06/2024 | FUA1 | FTA1 | TUA1 |
| 16/06/2024 | FUA2 | FTA2 | TUA2 |
| 02/07/2024 | FUA3 | FTA3 | TUA3 |
| 17/07/2024 | FUA4 | FTA4 | TUA4 |
| 03/08/2024 | FUA5 | FTA5 | TUA5 |
| 21/08/2024 | FUA6 | FTA6 | TUA6 |
| 01/09/2024 | FUA7 | FTA7 | TUA7 |
| 19/09/2024 | FUA8 | FTA8 | TUA8 |
| 08/10/2024 | FUA9 | FTA9 | TUA9 |
| 23/10/2024 | FUA10 | FTA10 | TUA10 |
| 07/11/2024 | FUA11 | FTA11 | TUA11 |
| 23/11/2024 | FUA12 | FTA12 | TUA12 |

**Table S2. AC:TP mass ratios used in the removal of COD from FUA samples.**

| Sample Code | Animal Charcoal (AC) | Tissue Paper (TP) | Mass ratio (AC:TP) |
|---|---|---|---|
| FUA2 | 1.0 g | 0 g | 1:0 |
| FUA2 | 0.5 g | 0 g | 0.5:0 |
| FUA2 | 0.25 g | 0.25 g | 0.25:0.25 |
| FUA2 | 1.0 g | 0.25 g | 1:0.25 |
| FUA2 | 1.0 g | 0.5 g | 1:0.5 |
| FUA6 | 0 g | 1.0 g | 0:1 |
| FUA6 | 0.5 g | 0.5 g | 0.5:0.5 |
| FUA6 | 1.0 g | 0.5 g | 1:0.5 |
| FUA12 | 1.0 g | 1.0 g | 1:1 |

| | | | |
|---|---|---|---|
| FUA12 | 0.25 g | 0 g | 0.25:0 |
| FUA12 | 1.0 g | 0.5 g | 1:0.5 |
| FUA12 | 1.0 g | 0.25 g | 1:0.25 |

## S2. Additional descriptive statistics and correlations

**Table S3. Baseline wastewater characteristics (mean ± SD; n = 12 per area).**

| Parameter | Fuel Unloading area (FUA) | Fuel Tanks area (FTA) | Turbine Units area (TUA) |
|---|---|---|---|
| pH | 7.00 ± 0.63 | 6.99 ± 0.56 | 7.25 ± 0.41 |
| TDS (mg/L) | 196.02 ± 32.42 | 247.05 ± 47.91 | 150.92 ± 24.20 |
| TSS (mg/L) | 671.17 ± 58.51 | 930.08 ± 51.95 | 574.75 ± 44.89 |
| Turbidity (NTU) | 641.67 ± 43.45 | 818.33 ± 43.03 | 570.83 ± 56.48 |
| COD (mg/L) | 2329.00 ± 258.30 | 3458.92 ± 214.12 | 1916.08 ± 118.61 |
| Ammonia (mg/L) | 0.68 ± 0.39 | 1.22 ± 0.46 | 0.42 ± 0.30 |
| Nitrite (mg/L) | 1.62 ± 0.68 | 2.11 ± 0.74 | 0.31 ± 0.20 |
| Nitrate (mg/L) | 11.04 ± 2.65 | 11.23 ± 4.50 | 6.63 ± 2.70 |
| Phosphate (mg/L) | 1.68 ± 0.68 | 1.70 ± 1.13 | 1.78 ± 1.12 |
| Oil & Grease (mg/L) | 349.35 ± 38.74 | 518.81 ± 32.11 | 288.96 ± 16.02 |

**Table S4. Pearson correlation coefficients (r) among key pre-treatment indicators (n = 36).**

| Variables | COD | TSS | Turbidity | Filtration Time |
|---|---|---|---|---|
| COD | 1.000 | 0.995 | 0.955 | 0.900 |
| TSS | 0.995 | 1.000 | 0.981 | 0.905 |
| Turbidity | 0.955 | 0.981 | 1.000 | 0.883 |
| Filtration Time | 0.900 | 0.905 | 0.883 | 1.000 |

*Note: All correlations are statistically significant (two-tailed; $p < 0.001$; $n = 36$).*

**Figure S1. Correlation matrix heatmap of pollution indicators (pre-treatment).**

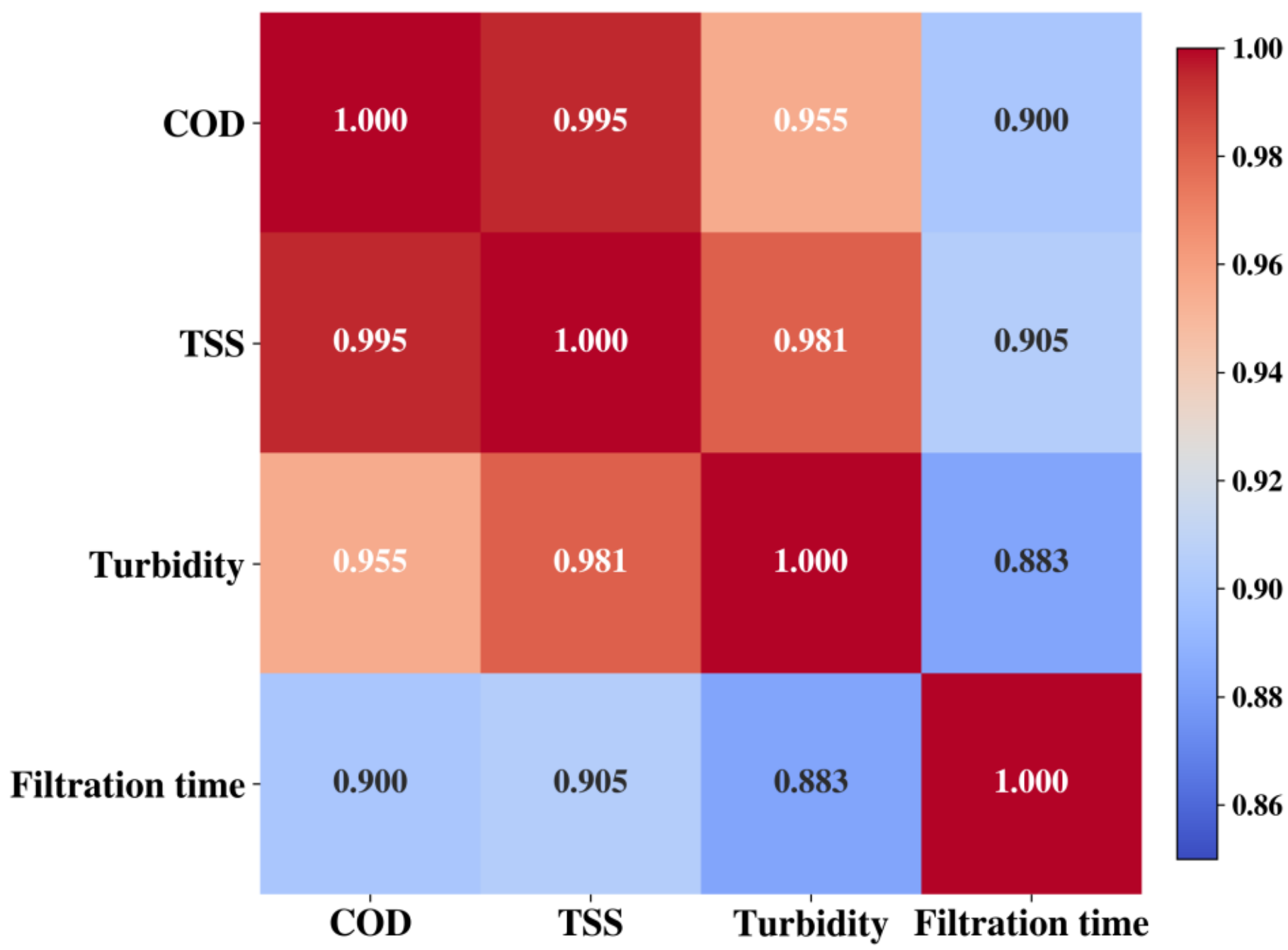


## S3. Dosage optimization statistics

**Table S5. One-way ANOVA for the effect of AC:TP dosage on COD removal efficiency (FUA dose-screening).**

| Source | Sum of Squares | df | Mean Square | F-value | Sig. (p) | Eta Squared |
|---|---|---|---|---|---|---|
| Between Groups | 4200.93 | 8 | 525.12 | 14.14 | 0.011 | 0.966 |
| Within Groups | 148.53 | 4 | 37.13 | | | |

*Note: One-way ANOVA; df = (8, 4); significance at p < 0.05.*

**Text S1. Key Tukey post-hoc pairwise comparisons for dosage optimization.**

- 1.0g AC : 0.5g TP vs. TP Only (0g AC : 1.0g TP): The difference was +63.38% ($p = 0.008$). This supports that tissue paper alone cannot effectively remove organic pollution; animal charcoal is necessary for adsorption.

- 1.0g AC : 0.5g TP vs. Low AC (0.25g AC): The optimal dose was +43.77% better ($p = 0.030$). This shows that enough carbon is required to provide enough surface area for the oil to stick to.
- 1.0g AC : 0.5g TP vs. High TP (1.0g AC : 1.0g TP): The difference was only 3.41% ($p = 0.999$, not significant). This result is very important. It means that adding more tissue paper (increasing from 0.5g to 1.0g) did not improve significantly.

**Figure S2. Effect of AC:TP dosage on COD removal efficiency (FUA dose-screening).**

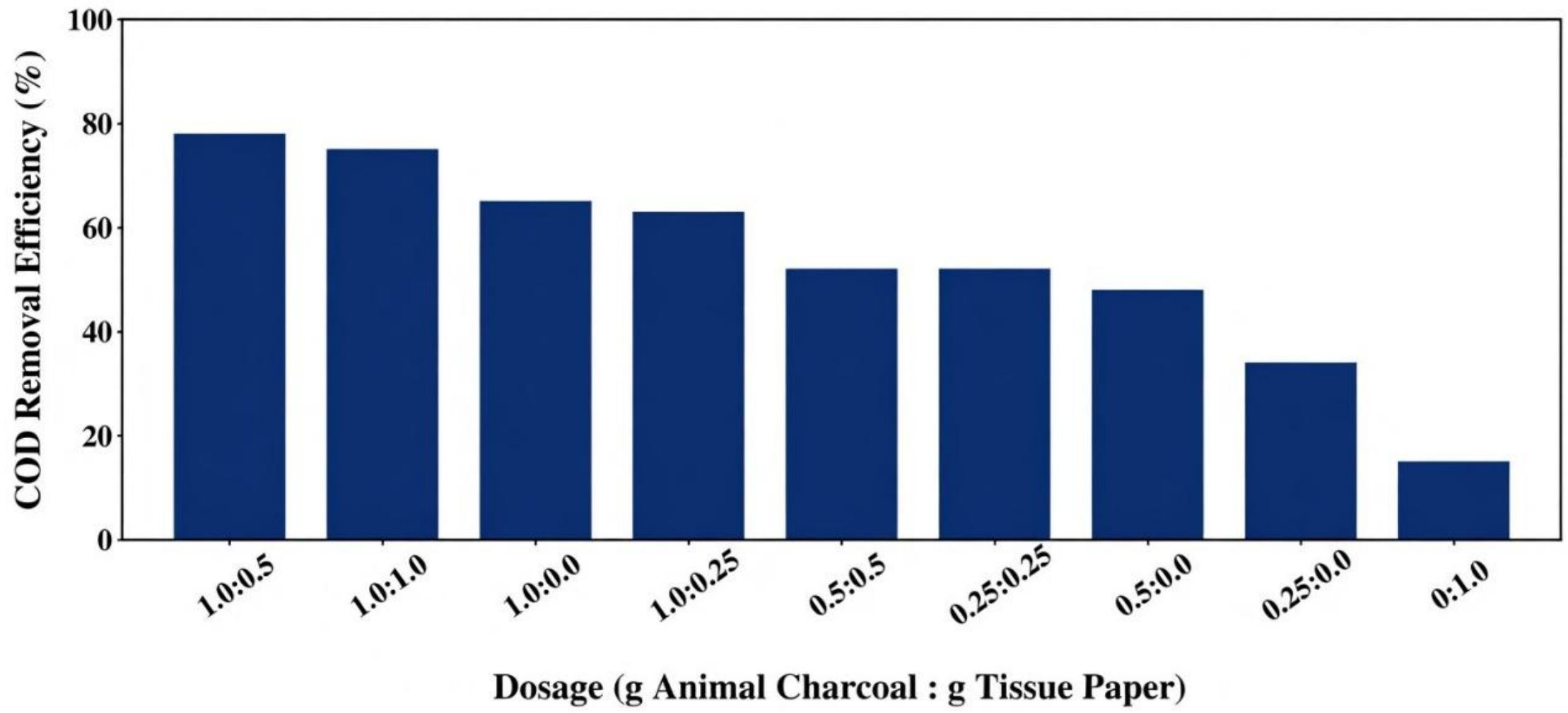


## S3. Treatment performance statistics at the optimal dose (1.0 g AC : 0.5 g TP)

**Figure S3. Comparison of pre- and post-treatment parameters (mean ± SD) at the optimal dose.**

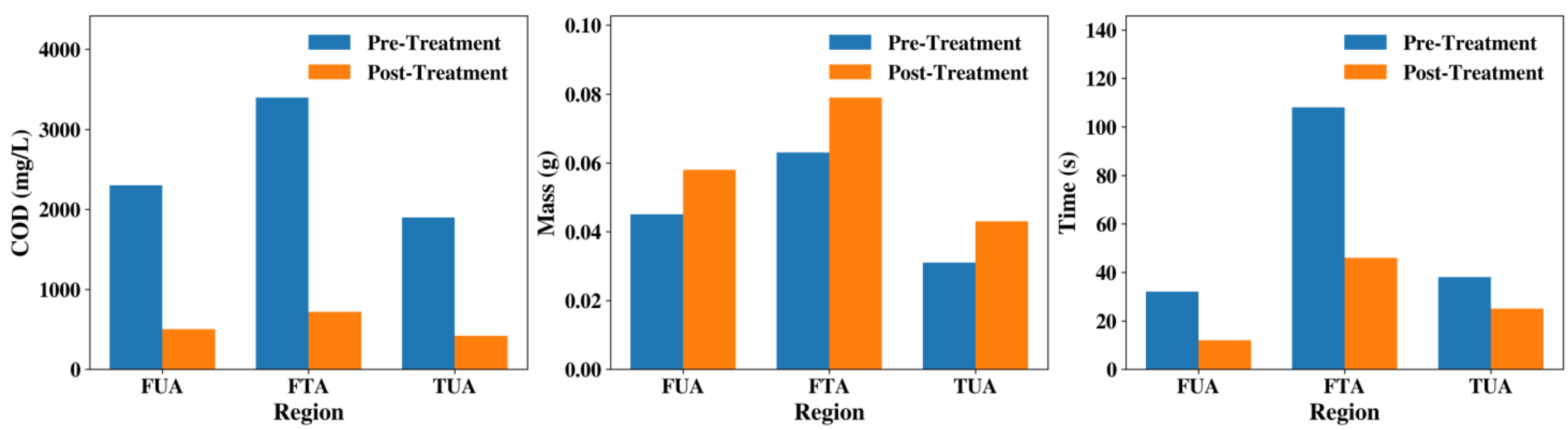

**Table S6. Paired-samples t-test results comparing pre- vs post-treatment values (all areas combined).**

| Variable | Mean Diff | t-statistic | p-value | Cohen’s d | Result |
|---|---|---|---|---|---|
| COD | 1675.2 | 11.06 | < 0.001 | 3.47 | Significant Reduction |
| Retained Solids | -0.012 | -11.10 | < 0.001 | 0.70 | Significant Increase |
| Filtration Time | 35.8 | 3.94 | 0.004 | 1.06 | Significant Reduction |

*Note: Mean Diff is calculated as pre-treatment minus post-treatment; negative values indicate an increase after treatment (e.g., retained solids).*

**Table S7. One-way ANOVA testing the effect of operational area on removal efficiency at the optimal dose.**

| Source | Sum of Squares | df | Mean Square | F-value | Sig. (p) | Eta Squared |
|---|---|---|---|---|---|---|
| Between Groups | 0.42 | 2 | 0.21 | 0.066 | 0.937 | 0.021 |
| Within Groups | 19.15 | 6 | 3.19 | | | |
